# Parental education, Family Health Climate and accelerometer-based measured physical activity and sedentary behavior of primary school-aged children

**DOI:** 10.3389/fpubh.2024.1385703

**Published:** 2024-11-06

**Authors:** Alexandra Ziegeldorf, Nina Hottenrott, Johanna Moritz, Petra Wagner, Hagen Wulff

**Affiliations:** ^1^Institute for Exercise and Public Health, Faculty for Sports Science, Leipzig University, Leipzig, Germany; ^2^Department of Sports Pedagogy, Faculty for Sports Science, Leipzig University, Leipzig, Germany

**Keywords:** parental education, Family Health Climate, moderate-to-vigorous physical activity, sedentary behavior, children

## Abstract

**Introduction:**

Sociodemographic factors such as parental education level (ED) influence the physical activity (PA) and sedentary behavior (SED) of primary school-aged children. In this context, family factors, such as the physical activity-related Family Health Climate (FHC_PA_), are relevant. However, the effect of FHC_PA_ on the interaction between ED and children’s activity behavior has not yet been investigated. Therefore, this study aimed to analyze the mediating effect of FHC_PA_ on the relation between parental ED and children’s device-based measured PA and SED.

**Methods:**

A total of 94 children and their parents participated in the study. Questionnaires were used to assess parental ED and FHCPA. Children’s moderate to vigorous physical activity (MVPA) and SED were measured using accelerometers. Bivariate correlations were conducted to investigate associations between parental ED and MVPA/SED/FHC_PA_. Mediation analyses were used to investigate the role of FHCPA in the association between maternal and paternal ED and children’s MVPA/SED. Results indicate a small correlation between maternal ED and FHC_PA_ for the total sample (ρ = 0.318, *p* < 0.001) and a medium correlation for girls only (ρ = 0.570, *p* < 0.001). Mediation analyses showed no significant mediation effect. However, there was a significant direct association when considering FHC_PA_ in the relation between higher maternal ED and SED in girls compared to lower ED.

**Discussion:**

Future research should examine more complex models to further develop and refine to facilitate the derivation of more effective recommendations for health prevention programs, particularly for mothers and girls.

## 1 Introduction

Energy balance-related behaviors, including nutrition, sleep, physical activity (PA), and a sedentary lifestyle play a significant role in the development of health and the onset of chronic conditions, such as overweight and obesity (1). Unfortunately, international studies and reports continue to show that children’s PA levels worldwide are too low and many children do not meet the recommended guidelines of 60 minutes (min) moderate to vigorous physical activity (MVPA) per day (2). Previous studies showed that girls are even more affected than boys (3, 4). The factors contributing to the disparity in PA involvement between genders are poorly understood (5). Besides various other settings (e.g., childcare facilities, school, community and peer groups), primary barriers preventing children, especially girls, from participating in PA can mainly be found in the family environment (e.g., sociodemographic factors, PA modeling, beliefs, attitudes and knowledge of family members, parental emotional and logistical support, household practices) (5, 6).

Various family factors, such as parental socioeconomic status (SES) indicators, parental beliefs, expectations, and social support, greatly impact children’s PA behavior (6–8). Particularly during the COVID-19 pandemic, where lockdowns and stay-home recommendations led children to spend even more time in the family environment, studies showed that parental encouragement and parental co-participation are highly associated with children’s PA behaviors (9).

A substantial body of research suggests the significant influence of the family context on children’s health-related behaviors. Traditional research to investigate family influences on children’s health-related behavior such as PA is unidirectional and focused on the parent–child subsystem (8, 10). However, family influences are far more complex and interact on different levels (11). In addition to individual parental factors (e.g., own PA values or intrinsic motivation) and dyadic influences between parents and children (e.g., parenting or role modeling), there are socialization dynamics at the whole family level that also influence the development and maintenance of a healthy lifestyle. Influencing factors at family-as-a-whole-level include i. a. family level behavior-specific cognitions and motivation. It is assumed that factors at this level are indirectly related to children’s health-related behaviors (11). A relevant construct in this context is the Family Health Climate (FHC). The FHC encompasses the shared perceptions and cognitions within the family, which concern a healthy lifestyle (12). It refers to the experience and evaluation of common health-related activities, routines, and topics in everyday family life (12). Evidence about the association of FHC and children’s PA has been limited. Existing results in this research field showed positive associations (13). However, existing results are based on self-reported data. To the best of our knowledge, associations with device-based measured PA are not present.

Various family factors influence each other in different ways. Parent and family socioeconomic characteristics (e.g., education, family income, occupation) are considered as shaping factors for parents’ beliefs and behaviors in general (e.g., efficacy beliefs, general and specific values or gender-role stereotyped beliefs), the family climate and general child-rearing styles, parents’ role modeling behavior and child-specific beliefs (e.g., perceptions of child’s abilities and interests, encouragement of activities) which in turn affect children’s beliefs, values, motivation and behavior (14). Thus, parent’s socioeconomic characteristics (e.g., education) may be shaping behavior-specific characteristics at the family level such as the physical activity related FHC (FHC_PA_), which is in turn related to important health-related behaviors of children (e.g., PA). Therefore, it can be assumed that parental educational level (ED) could influence children’s PA, which might be mediated by the FHC_PA_. Empirical evidence to support these theoretical assumptions is lacking.

Therefore, the aim of this study is twofold. Firstly, this study aimed to examine the association between parental ED, children’s MVPA, sedentary behavior (SED) and FHC_PA_. The second aim of this study is to investigate the mediating role of the FHC_PA_ in the association between parental ED and children’s MVPA/SED.

## 2 Materials and methods

### 2.1 Design, enrollment and participation

The data used for this analysis was obtained from the collaborative project “Family^+^” (‘Family^+^ - Living healthily together in family and school’/‘Familie^+^- Zusammen gesund leben in Familie und Schule). “Family^+”^ is a community-based, participatory obesity prevention program for primary school children (third and fourth grade) in German municipalities, developed, implemented, and evaluated by the University of Konstanz, Leipzig University, the Technical University of Munich and platform nutrition and physical activity (peb e.V.) and funded by the Federal Ministry of Health, Germany.

Before participating in the study, all parents or legal guardians of children were informed about the content and the measurement procedures of the study and written informed consent was obtained. A total sample of 453 agreed to participate in the program evaluation (questionnaire at three measurement points). Within this sample, 94 also agreed to participate in device-based data collection. For this secondary analysis, only baseline data from the program evaluation was used. Data was collected in August and September 2021. Approval for this study was obtained in 2021 from the Ethics Committee of the University of Konstanz (protocol code 417/2021. Date of approval: 10 January 2021).

### 2.2 Data collection procedures

A paper-pencil questionnaire for parents was used to collect sociodemographic and socioeconomic data and FHC_PA_. Accelerometers were used to measure children’s MVPA and SED. Questionnaires and accelerometers were handed out, explained, and reinserted on site by trained research staff from the faculty of sports science of the University of Leipzig.

### 2.3 Socioeconomic data

Maternal and paternal education (ED) were assessed using eight categories covering the most common educational degrees in Germany (“lower secondary school certificate,” “intermediate secondary school leaving certificate,” “Polytechnic secondary school/10th grade,” “advanced technical secondary school/vocational extension certificate,” “general/subject-related higher education entrance qualification/A-levels,” “other school-leaving qualification,” “leave school without qualifications” and “no school-leaving qualification (at the moment)”). The CASMIN-classification (15) was used to categorize parental ED into three groups: lower ED, moderate ED, and higher ED.

### 2.4 Physical activity related Family Health Climate

FHC_PA_ was assessed using the FHC_PA_-Scale from a validated questionnaire (12). The FHC_PA_-Scale consists of three subscales with a total of 14 items, rated on a 4-point Likert-type scale (1 = ‘definitely false’, 2 = ‘rather false’, 3 = ‘rather true’, 4 = ‘definitely true’). The subscales are composed as follows: value (e.g., “In our family … we make a point of being physically active during daily life.”), cohesion [e.g., “(...) we find it very pleasant to be physically active together.”] and information [e.g., “(...) we explicitly look for the latest information on physical activity and exercise to stay up to date.”]. A mean score of all FHC_PA_ items was calculated. The internal consistency for the FHC_PA_-Scale in this study was high (*α* = 0.836).

### 2.5 Moderate to vigorous physical activity (MVPA) and sedentary behavior (SED)

Children’s MVPA and SED were measured using Actigraph GT3X+ and wGT3X+ (2023 ActiGraph LLC, Pensacola, Florida, USA). ActiLife software v. 6.8.2 (released 2019, ActiGraph LLC, Pensacola, Florida, USA) was used for initialization and data processing. Accelerometers were worn on the non-dominant waist side (anterior axillary line) consistent for 7 days (waking hours). 15-s epoch length was used to capture short bouts of movement and a sampling frequency of 100 Hz was selected (16, 17).

### 2.6 Data preparation

Wear time validation (wear and non-wear time intervals) was defined by 90-min time windows for consecutive zero/non-zero counts and included 2-min intervals of non-zero counts with up/downstream 30-min consecutive zero counts windows for artifactual movement detection (18, 19). Valid datasets were defined by a wear time of at least 10 h per day for ≥ 4 days (17). Cut-points were defined for MVPA (2296 counts per minute) and SED (100 counts per min) (16, 17). Based on the measured individual daily values, a min/daily average was calculated for the respective week.

Missing data was imputed regarding recommendations of multiple data imputation (20). After checking that missing values were completely at random, as assessed by using the Little’s MCAR test and the visual inspection of the pattern of missing data (21), expectation maximization algorithm was used. Missing values (10.1% for device-based measured data and ranged between 14.9 and 23.4% for self-reported data) were imputed based on the existing valid data sets. The imputed values were analyzed using *t*-tests and no major differences were identified.

### 2.7 Data analyses

Statistical analyses were conducted on the imputed data set using IBM SPSS Statistics v. 27. Bivariate correlations (Spearman’s ρ) were used for analyzing the associations between parental ED and children’s MVPA/SED and FHC_PA_. According to Cohen (22), a Spearman’s ρ of 0.10 expresses a small correlation, 0.30 a medium correlation and 0.50 a strong correlation.

**Figure 1:**
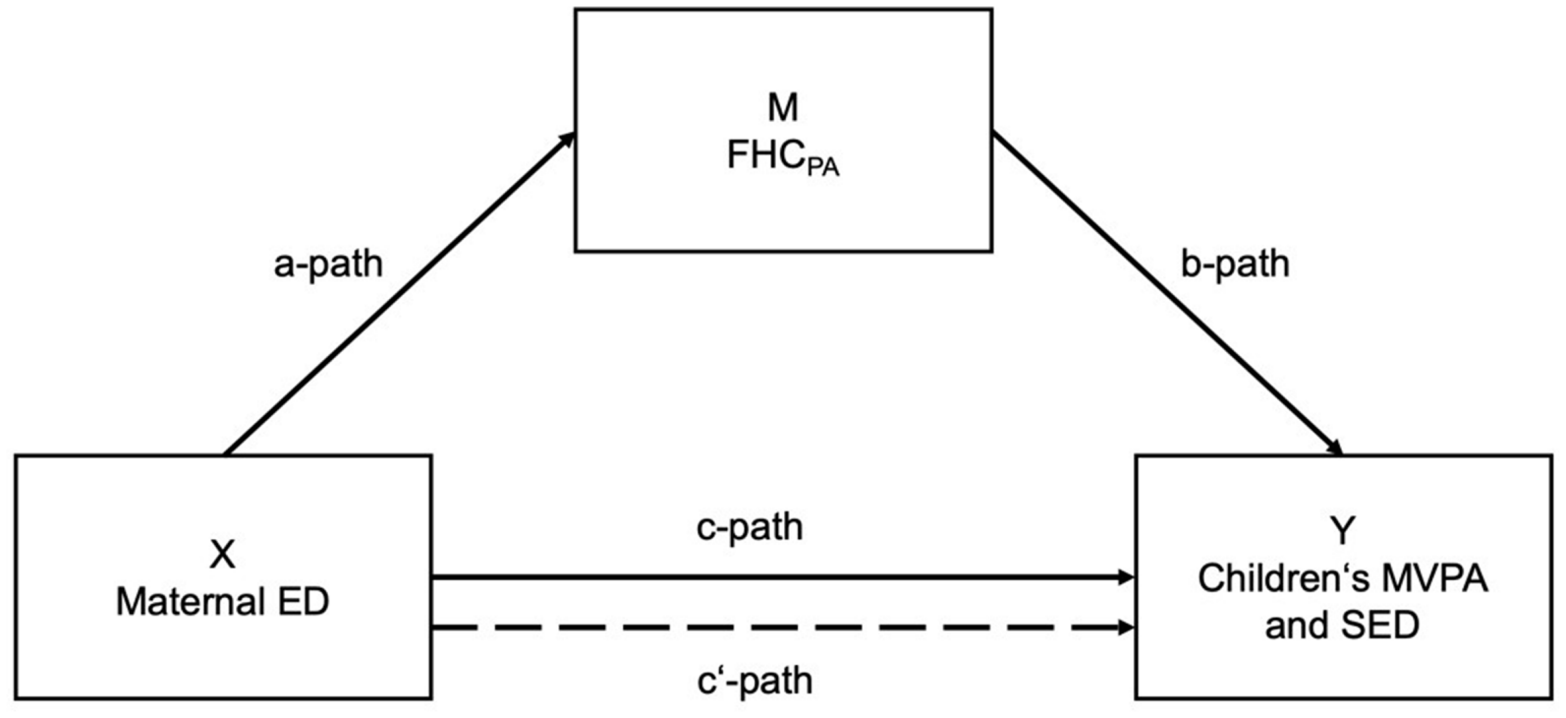
Mediation model of the association between maternal ED and children’s MVPA/SED (Model A). *Notes*: Predictor variable (X); outcome variable (Y); potential mediator variable (M); a-path: association between X and M; b-path: association between M and Y; c-path: total association between X and Y; c′-path: direct effect (mediated) X on Y.

**Figure 2:**
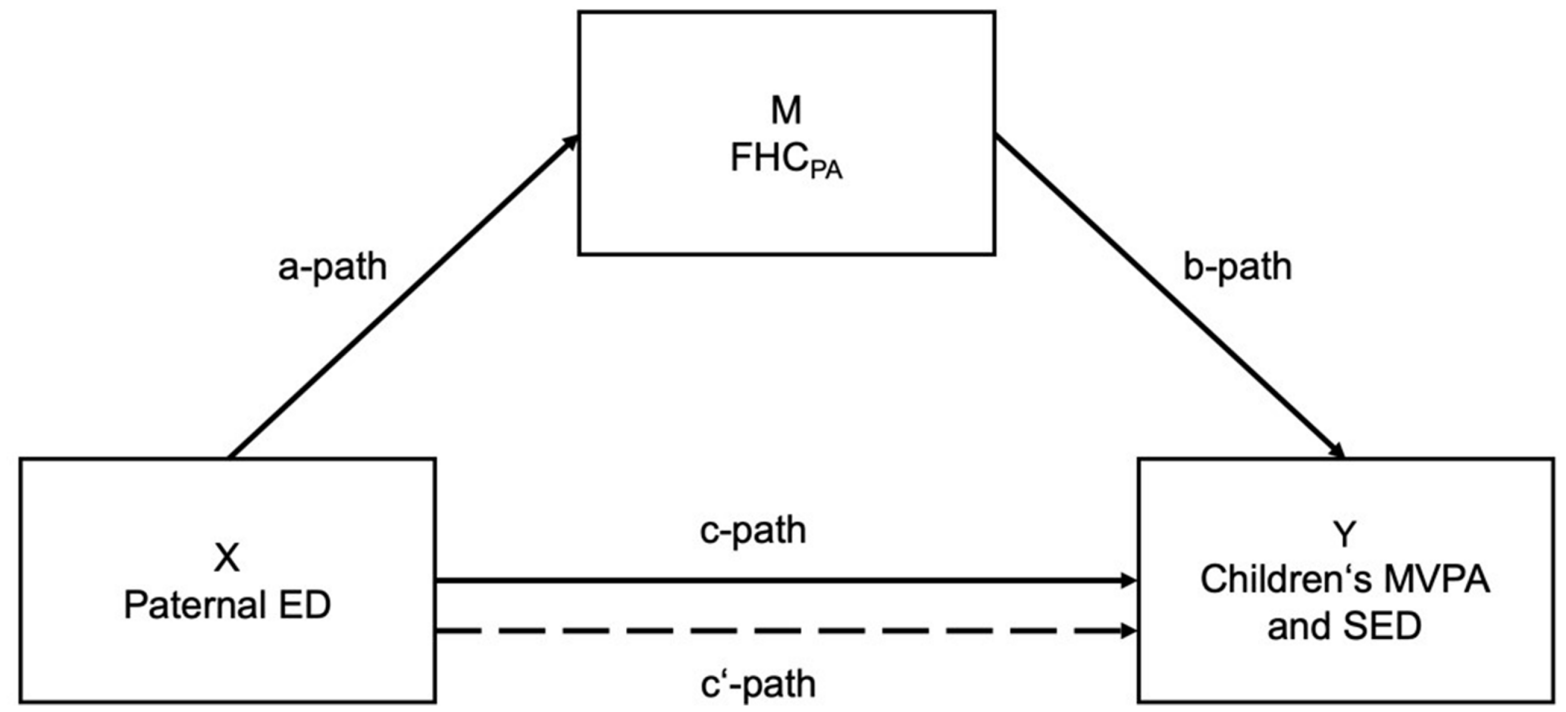
Mediation model of the association between paternal ED and children’s MVPA/SED (Model B). *Notes*: Predictor variable (X); outcome variable (Y); potential mediator variable (M); a-path: association between X and M; b-path: association between M and Y; c-path: total association between X and Y; c′-path: direct effect (mediated) X on Y.

Simple mediation analysis were used to calculate the potential mediating effect of FHC_PA_ on the association between parental ED and children’s MVPA and SED. Ordinary least squares regression was conducted using the PROCESS macro v4.0 (23). Linearity of relationships was checked by visual inspection (scatterplots). Indicator coding was used to describe the multicategorical variable of the parental ED [lower ED = reference category (RC)]. The hypothetical model was tested by 95% CI with 5,000 bootstrapped samples. Different mediation analyses were conducted for the association between FHC_PA_ [mediating variable (M)], maternal and paternal ED [predictor variables (X)], and children’s MVPA and SED [outcome variables (Y)]. Model A shows the pathways of calculations regarding the associations of maternal ED (Figure 1) and model B refers to the calculations regarding paternal ED (Figure 2).

A mediation effect is present when parental ED (maternal/paternal) affects children’s MVPA and SED (c-path) and FHC_PA_ (a-path), which itself might affect children’s MVPA and SED (b-path). Particularly, c’-path quantifies the direct effect of parental ED on children’s MVPA/SED adjusted for the mediator, while the product of a and b (a*b) quantifies the indirect effect of ED on children’s MVPA/SED through the mediator FHC_PA_ (Figures 1, 2).

Effects were considered to be statistically significant if the CI did not contain zero (*p* < 0.05).

## 3 Results

The data of 94 children (51.1% female) with a mean age of 9 (SD = ±1) years were included in the analysis. FHC_PA_ mean score was 2.57 (SD = ±0.48). Most parents reported a moderate ED (46.8% fathers, 43.6% mothers), followed by higher ED (42.6% fathers, 41.5% mothers) (Table 1). Descriptive analyses of the device-based measured data showed that children spent an average of 63.03 (SD = 21.62) min per day in MVPA and 483.49 (SD = 58.29) min in SED (Table 2). Consequently, 45.7% of the children (34.9% of boys, 65.1% of girls) did not reach WHO recommendations of 60 min MVPA per day (24).

**Table 1:** Sample characteristics (FHC_PA_ and parental ED).

		Total sample Mean (SD)	Mother % (*N*)	Father % (*N*)
	FHC_PA_	2.57 (0.48)		
Total sample (*N* = 94)	Lower ED		14.9 (14)	10.6 (10)
	Moderate ED		43.6 (41)	46.8 (44)
	Higher ED		41.5 (39)	42.6 (40)
Boys (*N* = 46)	Lower ED		13.0 (6)	8.7 (4)
	Moderate ED		43.5 (20)	45.7 (21)
	Higher ED		43.5 (20)	45.7 (21)
Girls (*N* = 48)	Lower ED		16.7 (8)	12.5 (6)
	Moderate ED		43.8 (21)	47.9 (23)
	Higher ED		39.6 (19)	39.6 (19)

**Table 2:** Descriptive analyses (children’s MVPA and SED).

	MVPA*Mean (SD)		SED *Mean (SD)	
	**Maternal ED**	**Paternal ED**	**Maternal ED**	**Paternal ED**
Total sample	63.03 (21.62)		483.49 (58.29)	
*Lower*	62.87 (16.12)	63.92 (19.34)	472.58 (38.76)	485.04 (63.85)
*Moderate*	57.38 (21.80)	60.95 (23.91)	484.49 (64.22)	488.66 (61.75)
*Higher*	69.02 (22.00)	65.09 (19.72)	486.34 (58.38)	477.41 (53.75)
Boys	68.59 (22.04)		478.27 (62.19)	
*Lower*	68.42 (20.74)	62.86 (25.00)	473.94 (53.10)	457.50 (45.39)
*Moderate*	62.23 (22.09)	70.47 (24.36)	484.42 (68.46)	495.08 (69.89)
*Higher*	75.01 (21.53)	67.81 (19.87)	473.41 (60.51)	465.41 (54.33)
Girls	57.69 (20.02)		488.49 (54.49)	
*Lower*	58.70 (11.37)	64.63 (17.22)	471.55 (27.81)	503.40 (71.36)
*Moderate*	52.77 (21.00)	52.26 (20.32)	484.56 (61.61)	482.79 (54.19)
*Higher*	62.71 (21.23)	62.08 (19.63)	499.96 (54.30)	490.67 (51.26)

*n* = 94 (*n* = 46 for boys and *n* = 48 for girls). *Average measured in minutes per day.

Results of the correlation analyses are shown in Table 3. A medium correlation was found between maternal ED and FHC_PA_ in the total sample (ρ = 0.318, *p* < 0.001) as well as a strong correlation between these variables for girls (ρ = 0.570, *p < 0.*001). Correlations between paternal ED and FHC_PA_ scores did not show statistical significance.

**Table 3:** Spearman correlation coefficients (ρ) between children’s MVPA/SED/FHC_PA_ and parental ED.

		FHC_PA_	Parental ED	
			Maternal	Paternal
Total sample	MVPA	0.026	0.185	0.085
	SED	0.035	0.077	−0.018
	FHC_PA_		0.318*	0.061
Boys	MVPA	0.129	0.209	0.011
	SED	0.048	0.059	0.135
	FHC_PA_		0.114	0.072
Girls	MVPA	−0.098	0.118	0.098
	SED	0.023	0.217	0.101
	FHC_PA_		0.570**	0.071

Significant results are indicated in bold. *p > 0.05, **p > 0.001.

### 3.1 Parental ED and children’s time spent in MVPA/SED (c-path)

Higher maternal and paternal ED were associated with higher children’s MVPA for total sample and boys compared to RC. In addition, higher maternal ED was associated with higher children’s MVPA for girls too. Moderate maternal ED was associated with children spending less time in MVPA for the total sample, girls and boys compared to RC (Tables 4, 5).

**Table 4:** Mediation effect of FHC_PA_ on association between maternal ED and children’s MVPA/SED.

		Maternal ED	c-path^a^(95% CI)	a-path^b^ (95% CI)	b-path^c^ (95% CI)	Indirect effect(a * b) (95% CI)	c’-path^d^(95% CI)
t	MVPA	Moderate*	−5.482 (−16.697; 5.733)	**0.382 (0.142; 0.622)**	2.955 (−6.629; 12.539)	1.128 (−2.698; 5.091)	−6.610 (−19.515; 6.295)
		Higher*	6.154 (−5.211; 17.518)	**0.449 (0.212; 0.687)**		1.327 (−3.183; 5.939)	4.827 (−6.629; 12.539)
	SED	Moderate*	11.916 (−17.459; 41.291)	**0.382 (0.142; 0.622)**	−3.396 (−36.246; 29.455)	−1.296 (−15.596; 10.758)	13.212 (−23.280; 49.704)
		Higher*	13.765 (−14.694; 42.224)	**0.449 (0.212; 0.687)**		−1.525 (−19.685; 11.514)	15.290 (−17.810; 48.390)
m	MVPA	Moderate*	−6.184 (−27.500; 15.131)	0.402 (−0.095; 0.900)	9.900 (−3.063; 22.862)	3.983 (−2.130; 12.325)	−10.168 (−31.674; 11.339)
		Higher*	6.592 (−14.600; 27.784)	0.281 (−0.220; 0.782)		2.781 (−2.182; 10.709)	3.811 (−14.392; 22.014)
	SED	Moderate*	10.483 (−46.937; 67.902)	0.402 (−0.095; 0.900)	5.693 (−40.144; 51.529)	2.291 (−20.479; 22.909)	8.192 (−59.157; 75.542)
		Higher*	−0.534 (−56.010; 54.941)	0.281 (−0.220; 0.782)		1.599 (−18.938; 16.716)	−2.134 (−61.547; 57.280)
f	MVPA	Moderate*	−5.937 (−18.757; 6.883)	**0.353 (0.097; 0.610)**	−7.182 (−24.237; 9.874)	−2.537 (−9.415; 3.096)	−3.399 (−18.882; 12.083)
		Higher*	4.012 (−9.273; 17.296)	**0.613 (0.394; 0.833)**		−4.404 (−14.681; 5.341)	8.416 (−7.991; 24.823)
	SED	Moderate*	13.005 (−21.899; 47.908)	**0.353 (0.097; 0.610)**	−31.790 (−83.902; 20.323)	−11.232 (−33.415; 5.247)	24.237 (−20.860; 69.333)
		Higher*	28.404 (−4.955; 61.762)	**0.613 (0.394; 0.833)**		−19.495 (−53.172; 8.991)	**47.899 (2.040; 93.757)**

Regression coefficients (B); * Lower ED served as RC. Significant results are indicated in bold. ^a^ Total association between maternal ED and children’s MVPA/SED. ^b^ Association between maternal ED and FHC_PA._. ^c^ Association between FHC_PA_ and children’s MVPA/SED. ^d^ Direct association between maternal ED and MVPA/SED after adjusting for mediator. t = total sample; m = male; f = female.

**Table 5:** Mediation effect of FHC_PA_ on association between paternal ED and children’s MVPA/SED.

		Paternal ED	c-path^a^(95% CI)	a-path^b^(95% CI)	b-path^c^(95% CI)	Indirect effect (a * b)	c’-path^d^(95% CI)
t	MVPA	Moderate*	−2.973 (−17.685; 11.738)	0.231 (−0.126; 0.588)	3.672 (−6.094; 13.438)	0.848 (−1.331; 4.646)	−3.821 (−19.207; 11.566)
		Higher*	1.171 (−13.087; 15.429)	0.218 (−0.152; 0.588)		0.800 (−1.291; 4.941)	0.371 (−14.002; 14.744)
	SED	Moderate*	3.618 (−42.614; 49.850)	0.231 (−0.126; 0.588)	0.095 (−28.182; 28.372)	0.022 (−8.714; 8.217)	3.596 (−42.350; 49.542)
		Higher*	−7.628 (−53.233; 37.977)	0.218 (−0.152; 0.588)		0.021 (−9.024; 7.309)	−7.649 (−52.935; 37.638)
m	MVPA	Moderate*	7.606 (−23.505; 38.718)	0.430 (−0.351; 1.212)	7.676 (−7.421; 22.774)	3.303 (−3.235; 17.049)	4.303 (−33.739; 42.345)
		Higher*	4.954 (−25.501; 35.410)	0.403 (−0.384; 1.190)		3.094 (−3.202; 17.095)	1.860 (−34.606; 38.326)
	SED	Moderate*	37.576 (−23.957; 99.109)	0.430 (−0.351; 1.212)	3.896 (−35.803; 43.595)	1.676 (−21.464; 24.157)	35.899 (−28.711; 100.510)
		Higher*	7.914 (−50.338; 66.167)	0.403 (−0.384; 1.190)		1.570 (−22.325; 20.977)	6.344 (−56.663; 69.351)
f	MVPA	Moderate*	−12.371 (−30.171; 5.429)	0.096 (−0.294; 0.486)	−2.275 (−17.094; 12.544)	−0.219 (−3.615; 3.020)	−12.152 (−31.073; 6.768)
		Higher*	−2.545 (−20.644; 15.555)	0.098 (−0.329; 0.524)		−0.222 (−3.643; 3.437)	−2.275 (−17.094; 12.544)
	SED	Moderate*	−20.602 (−88.960; 47.755)	0.096 (−0.294; 0.486)	−10.220 (−55.014; 34.573)	−0.984 (−13.414; 4.957)	−19.618 (−87.063; 47.826)
		Higher*	−12.728 (−81.454; 55.998)	0.098 (−0.329; 0.524)		−0.999 (−13.890; 6.237)	−11.729 (−79.197; 55.739)

Regression coefficients (B); * Lower ED served as RC. ^a^ Total association between paternal ED and children’s MVPA/SED. ^b^ Association between paternal ED and FHC_PA._^c^ Association between FHC_PA_ and children’s MVPA/SED. ^d^ Direct association between paternal ED and MVPA/SED after adjusting for mediator. t = total sample; m = male; f = female.

Moderate maternal and paternal ED were associated with more time in SED in the total sample and boys in comparison to RC. Higher maternal ED was associated with more time spent in SED for the total sample and girls in comparison to RC, whereas higher paternal ED was associated with less time in SED than RC. Largest difference in model A was found in girl’s SED for higher maternal ED compared to RC. In model B children’s time spent in SED for boys showed the largest difference for moderate ED compared to RC.

### 3.2 Association between parental ED and FHC_PA_ (a-path)

The association between parental ED and FHC_PA_ showed that a higher ED was associated with a higher FHC_PA_. The association was significant for maternal ED for the total sample and girls.

### 3.3 Association between FHC_PA_ and MVPA/SED (b-path)

The association between FHC_PA_ and children’s MVPA showed that a higher FHC_PA_ was associated with children’s increased time in MVPA for the total sample and boys and a higher FHC_PA_ was associated with children’s decreased time in MVPA for girls.

As shown in Tables 4, 5, a higher FHC_PA_ was associated with children’s increased time spent in SED among boys and with a decreased time spent in SED among girls in both models. Model A showed also that a higher FHC_PA_ score was associated with a decreased time spent in SED for the total sample whereas in model B the association was inverse.

### 3.4 Mediation effect (a*b) (ED, FHC_PA_, MVPA/SED)

Mediation effects of FHC_PA_ on the association between maternal or paternal ED and children’s MVPA have not occurred. Also, the indirect effects of FHC_PA_ on the difference in children’s time spent in SED according to parental ED were not statistically significant (Tables 4, 5).

### 3.5 Direct association between paternal ED and MVPA/SED after adjusting for mediator (c’path)

The differences in children’s MVPA according to parental ED became not significant after including the mediator for the total sample, girls, and boys (Tables 4, 5). However, the differences in children’s MVPA according to parental ED changed after including the mediator. Specifically, the time spent in MVPA increased for moderate maternal ED compared to RC and the time spent in MVPA decreased for higher maternal ED compared to RC for total sample and boys. Whereas for girls the time spent in MVPA increased for higher maternal ED and the time spent in MVPA decreased for moderate maternal ED compared to RC. According to paternal ED, boys’ time spent in MVPA decreased for both moderate ED and higher ED compared to RC.

As presented in Table 4, the difference in girls’ SED, according to maternal ED, became significant after including the mediator for higher ED compared to RC. All other differences in SED related to parental ED remained non-significant. Time spent in SED increased for the total sample and girls for both, higher and moderate maternal ED, compared to RC. More specifically, the differences in girls’ SED nearly doubled for moderate and higher ED, whereas for higher and moderate paternal ED the differences in boys’ and girls’ SED decreased.

## 4 Discussion

In line with other recent results, we also found gender differences, with boys spending more time in MVPA and less time in SED than girls (3, 4). To gain a better understanding of association between important factors influencing children’s PA, the first aim of this study was to examine the association between maternal and paternal ED and FHC_PA_/MVPA/SED in primary school age.

Results showed significant positive correlation between maternal ED and FHC_PA_ in girls (*ρ* = 0.570, *p* < 0.001). This contrasts with the results reported by Niermann et al. (13), who found no significant differences in FHC_PA_ between lower vs. higher maternal level of education [FHCagg-PA: *t*(189) = 0.39, *p* = 0.70]. However, the findings from Niermann et al. (13) relate to adolescents, which limits the direct comparability of the results - mainly because the influence of parents is greater at primary school age than in adolescence, where the influence of friends and the peer group becomes more important (25). Possible explanations for the positive correlations in this study may related to allocative efficiency hypothesis, which posits that individuals with higher education are more adept at processing health information and thus higher educational attainment is associated with enhanced health knowledge, which in turn leads to healthier lifestyle choices. However, research findings on this topic are heterogeneous (26, 27). Furthermore, it has been found that resources to improve skills and traits, such as cognitive and problem-solving abilities concerning health topics, depend on ED (28). In particular, skills related to general health and knowledge about health topics are more frequently found in higher ED (28).

Significant correlations between FHC_PA_ score and education appeared only on the maternal side (Table 3). This could result from the fact that families in which the mothers have a higher ED also tend to exhibit healthier behaviors overall. And since mothers are more responsible for the health-related contexts (e.g., nutrition, organizing trips, etc.) in everyday family life, they also transfer their health related views, values and beliefs into their decisions and activities for their family life (29). Even if both parents influence the PA of their children, gender-specific differences can be observed. For example, the mother as a role model tends to influence the behavior of girls, while boys are more likely to follow their fathers as role models. Thus, the mother does influence health-related behaviors in the everyday family life of boys and girls. However, she seems to influence girls’ behaviors more than those of the boys (30, 31).

Regarding the association between parental ED and children’s SED, current studies predominantly showed negative associations, indicating that children from families with low parental education were more likely to spend at least 2 h of screen time per day than children with highly educated parents (6, 32). The results of our study are inconsistent concerning parental and children’s sex. While a higher maternal ED, compared to lower maternal ED, was associated with more time spent in SED in general, especially for girls, where significance was also observed, a higher paternal ED tended to be associated with less time spent in SED, particularly for boys. Possible explanations could include the educational trajectories of children from families with higher levels of education (33). Higher education parents may emphasize and support their children’s school success (34, 35), especially maternal education compared to paternal education showed a stronger relationship with children’s academic achievement (36). Higher forms of schooling usually also mean a higher workload with school tasks (e.g., higher amount of homework, longer school days) for the children and, conversely, more time spent sedentary (37).

As pointed out in the limitations, almost all studies focusing on children’s sedentary behavior assess only screen time instead of the overall daily time spent sedentary, which makes it difficult to compare results.

Results also showed a positive relation between maternal ED and MVPA for total sample, boys, and girls. Even if these results are in line with a few other findings in this field of research (38, 39), reviews predominantly show no (40) or negative associations between parental educational level and children’s PA, especially for highly educated mothers (6, 41).

The inconsistency in this association supports the assumption of higher complexity and underlining mediating factors particularly at the family level. For a better understanding in this respect, the second aim of this study was to examine the mediating role of the FHC_PA_ on the association of parental ED and children’s MVPA/SED (Figures 1, 2).

Against our expectations, results of this study do not indicate a mediating effect of the FHC_PA_ on the association between parental ED and children’s MVPA and SED. These results are in line with other results regarding family support, indicating an independent association with participants’ PA behavior, rather than mediating the influence of parental factors on children’s PA (42). Also, a study investigating the mediating effect of specific parenting practices on the relation between parental educational level and the screen time of primary school-aged children did not find clear evidence (43). There are only results avoiding negative role modeling concerning TV-time, but no other parenting practices, to mediate the inverse association between family SES and children’s screen-time (43).

However, in this study changes in these associations were recognized after the inclusion of FHC_PA_. This is particularly evident in the relation between maternal ED and SED/MVPA in girls, where the differences increased when taking the FHC_PA_ as a potential mediator into account. The results regarding children’s MVPA, which increases when FHC_PA_ is included as a mediator in this association, are consistent with the hypothesised causality of the underlying theoretical framework (14). Contrary to this theoretical causality, however, the same association was also found with regard to children’s SED. To better understand these controversial results, more and complex research is needed, which also takes other PA intensity ranges and further potential mediators into account (43). Comparative research, for example, demonstrated the mediating effect of motivation in the association between parental SES and children’s MVPA, suggesting that children’s motivation to engage in PA is influenced by their parents’ socioeconomic status, which in turn affects how much physical activity they actually engage in. The results indicate that as parental SES increases, children’s motivation to participate in physical activities also increases, leading to higher levels of MVPA (44).

Furthermore, in this context it is important to point out, that the association between FHC and activity behavior is complex and is also mediated by factors such as intrinsic motivation (13). However, children’s motivation itself as an important factor is also influenced by a variety of different aspects (13).

Therefore, future research should examine more complex modelling approaches (e.g., structural equation models) in order to take into account various influencing factors operating at different levels. Structural equation models could, for instance, help to analyze intricate associations between multiple variables simultaneously, enhance the modelling of causal relationships, and account for measurement errors to further develop and sharpen theoretical frameworks, particularly with regard to gender differences. This could facilitate the derivation of more effective and more appropriate recommendations in the area of pediatric health intervention and prevention (e.g., how exactly should the family be embedded).

Furthermore, longitudinal studies are also important for understanding the development of children’s PA and SED in relation to family influences, particularly in the context of gender differences. Longitudinal studies allow for more comprehensive monitoring of lifestyle habits and dynamic interactions within families and their impact on children’s activity patterns, thereby providing a more detailed understanding of the complexities involved in the development of children’s health-related behaviors.

### 4.1 Limitations

The current study has some limitations to point out, such as the lack of specific recommendations for sedentary behavior in children, the need for further research on different intensity ranges of PA, and the risk of bias.

The health-promoting effects of 60 min MVPA per day on average for children have been well-researched (45) and are clearly defined by the WHO (24). Therefore, MVPA was used to measure health-enhancing PA. In contrast, to the best of our knowledge, there are no specific recommendations regarding an exact amount of light, moderate and vigorous PA for children. Consequently, this study did not focus on these specific levels of PA intensity. To gain a more accurate understanding of children’s PA, further research and recommendations for light, moderate, and vigorous PA are necessary.

In addition, there is a lack of specific recommendations regarding children’s SED. To the best of our knowledge, there are no established guidelines for device-based measured SED in the literature. The recommendation that partly refers to SED is a maximum of 2 h screen time per day (46). Most studies only consider screen time when addressing SED. Screen time can be self-reported through a questionnaire, but it does not provide information about children’s overall SED. In this study, SED was measured using accelerometry, allowing a more accurate depiction of children’s time spent in SED. This enables a more comprehensive examination of children’s SED beyond just considering screen time. This makes it challenging to interpret SED in this study, since it was not defined only as screen time. Moreover, due to the lack of SED reference values in the literature, it is difficult to classify the results of this study. Furthermore, with regard to the health outcome, it is important to mention that one of the main factors is not necessarily the total SED amount, but the uninterrupted SED. Research has shown that, for example, interrupting prolonged sitting with a short active break can bring relevant health benefits (47–50). It would therefore also be interesting taken this factor under consideration in future analyses.

The absence of specific time values for reducing SED or increasing MVPA that have health-promoting effects on children makes it difficult to interpret the mediation analysis (the differences from path c to c’, i.e., the effect of a*b). As far as we know, the literature does not offer any guidance on how to interpret or classify decreases in SED and increases in MVPA values.

The accelerometer-based measurement of SED and MVPA in relation to FHC_PA_ in primary school children is a unique strength of this study, as (as far as we are aware) no other studies exist in this area. Device-based measurement enables precise and reproducible quantification of movement behavior and reduces the risk of recall bias that often occur with self-reported measurements (51), which also benefits the analyses in this study. However, the sample size in this study is relatively small because the analysis was conducted with already existing (baseline) data from the evaluation of a prevention program. Future studies should examine device-based measured activity data using larger samples to gain more insights in relation to this context.

Additionally, there might be a volunteer bias in reporting the FHC_PA_. Mothers and fathers might perceive FHC_PA_ differently and, therefore, evaluate it differently as well. Thus, to ensure comparability, it would be necessary to consider who is filling out the questionnaire. The subjective responses to the FHC_PA_ questionnaire may have led to a more positive assessment of the family conditions than what the reality provides.

## 5 Conclusion

Results of this study showed significant associations between FHC_PA_ score and maternal ED. The results also showed a positive relation between maternal ED and children’s MVPA. However, even though changes in the association between parental ED and children’s MVPA and SED occurred after the inclusion of FHC_PA_ as a mediating factor, the results do not suggest a mediating effect of FHC_PA_ in this context.

Some of the results are not consistent with the theoretically expected causal relationships. Future studies should examine other possible mediating factors to understand the mechanism through which parental education influences children’s PA and SED. Understanding these mechanisms can help to further develop framework models at a theoretical level and, based on this, inform pediatric health promotion strategies tailored to family dynamics at a practical level.

## Data Availability

The data analyzed in this study is subject to the following licenses/restrictions: data being used in ongoing project(s). Requests to access these datasets should be directed to the Institute for Exercise and Public Health, Faculty for Sports Science, University Leipzig, Germany. Email: igph@uni-leipzig.de.
